# Malaria health seeking practices for children, and intermittent preventive treatment in pregnancy in Wakiso District, Uganda

**DOI:** 10.4314/ahs.v21i4.28

**Published:** 2021-12

**Authors:** David Musoke, Rawlance Ndejjo, Solomon Tsebeni Wafula, Simon Kasasa, Jessica Nakiyingi-Miiro, Miph Boses Musoke

**Affiliations:** 1 Department of Disease Control and Environmental Health, School of Public Health, College of Health Sciences, Makerere University, Kampala, Uganda; 2 Department of Epidemiology and Biostatistics, School of Public Health, College of Health Sciences, Makerere University, Kampala, Uganda; 3 London School of Hygiene and Tropical Medicine, London, United Kingdom; 4 School of Sciences, Nkumba University, Entebbe, Uganda

**Keywords:** Health seeking behaviour, intermittent preventive treatment, malaria, children, pregnancy, Uganda

## Abstract

**Background:**

Timely health care among children with suspected malaria, and intermittent preventive treatment (IPTp) in pregnancy avert related morbidity and mortality in endemic regions especially in sub-Saharan Africa. Malaria burden has steadily been declining in endemic countries due to progress made in scaling up of such important interventions.

**Objectives:**

The study assessed malaria health seeking practices for children under five years of age, and IPTp in Wakiso district, Uganda.

**Methods:**

A structured questionnaire was used to collect data from 727 households. Chi-square and Fisher's exact tests were performed in STATA to ascertain factors associated with the place where treatment for children with suspected malaria was first sought (government versus private facility) and uptake of IPTp.

**Results:**

Among caretakers of children with suspected malaria, 69.8% sought care on the day of onset of symptoms. The place where treatment was first sought for the children (government versus private) was associated with participants' (household head or other adult) age (p < 0.001), education level (p < 0.001) and household income (p = 0.011). Among women who had a child in the five years preceding the study, 179 (63.0%) had obtained two or more IPTp doses during their last pregnancy. Uptake of two or more IPTp doses was associated with the women's education level (p = 0.006), having heard messages about malaria through mass media (p = 0.008), knowing the recommended number of IPTp doses (p < 0.001), and knowing the drug used in IPTp (p < 0.001).

**Conclusion:**

There is need to improve malaria health seeking practices among children and pregnant women particularly IPTp through programmes aimed at increasing awareness among the population.

## Introduction

Malaria remains a major cause of morbidity and mortality in endemic countries particularly in sub-Saharan Africa including Uganda. Children under five years of age and pregnant women are high risk groups regarding malaria infection due to their low immunity. For that reason, global malaria prevention efforts such as access to long-lasting insecticidal nets (LLINs) and Indoor Residual Spraying (IRS) have put much emphasis on these groups to reduce the occurrence of malaria among them^1^. There were 272,000 deaths due to malaria among children under five years in 2018, which accounted for 67% of total deaths worldwide^1^. In addition, approximately 125 million pregnant women globally are at risk of acquiring malaria infections annually, and 24% of these occur in sub-Saharan Africa^2^. Pregnant women affected by malaria infections can experience devastating effects including severe anemia, increased risk of preterm delivery, low birth weight, intrauterine death, stillbirth, miscarriages and maternal deaths^3^,^4^. The World Health Organization (WHO) recommends intermittent preventive treatment of malaria in pregnancy (IPTp) as part of the three-pronged strategic approach to malaria prevention and control for regions with high malaria endemicity and transmission including sub-Saharan Africa. The WHO policy also includes the use of LLINs, and effective management of clinical malaria and anemia including among children and pregnant women^5^. According to the policy, all pregnant women should receive IPTp during their antenatal care (ANC) visits which should commence early in the second trimester. Each IPTp dose with sulphadoxine-pyrimethamine (IPTp-SP) should be given at least a minimum 1 month apart but ensuring that at least 3 doses are given during pregnancy6. Due to potential mosquito resistance to insecticides used in IRS, and other challenges in malaria prevention in endemic countries including Uganda, IPTp is a key control strategy against malaria^1^. The WHO has also recommended integrated community case management (iCCM) of childhood illnesses including malaria1 which is carried out by Community Health Workers (CHWs) locally referred to as village health teams (VHTs) in Uganda.

Although iCCM and IPTp policies have been implemented in many sub-Saharan African countries including Uganda for many years, there is still low uptake^1^,^7^,^8^. The factors responsible for the low uptake of IPT include number and timing of ANC visits, low awareness, low education level^9^,^10^ and high costs^11^. Some systemic factors such as inadequate drug supply, poor skills and attitudes of health providers, shortage of health workers, and skewed access to ANC have also been cited^12^. On the other hand, iCCM has faced many challenges in Uganda and other malaria endemic countries mainly around financing which has not only affected training of CHWs but also availability of medicines and other supplies such as rapid diagnostic tests (RDTs)^1^. Factors such as deficiencies in the health system, long distance to nearest health care facilities (HCFs), and high cost of treatment services are also known to impede the uptake of most health services in Uganda including IPTp^13^. The success of treatment of children including under iCCM, and IPTp in malaria endemic countries is therefore dependent on various health systems building blocks including governance, financing, human resources, access to essential medicines, information systems and service delivery^14^.

Malaria is still endemic in most parts of Uganda where it is responsible for about 65% of maternal mortality, and 60% of spontaneous abortions among pregnant women^15^, as well as a prevalence of 19% among children under five years of age^16^. Although Uganda is implementing the IPTp policy, its uptake is still low. According to the 2016 Uganda Demographic and Health Survey (UDHS), only 16.8% of pregnant women had received three or more doses of IPTp-SP, 45.0% had received two or more doses, and 76.9% had received at least one dose^17^. Several challenges affect the uptake of malaria control interventions including IPTp, and prompt diagnosis and treatment among children and pregnant women in Uganda. These challenges include inadequate health care financing, limited coverage of HCFs, and irregular supply and underutilisation of drugs^18^. There is also low retention of staff at HCFs, as well as ineffective supervision, coordination and leadership^18^. Seeking IPTp is essential in mitigating adverse effects associated with maternal malaria, and also reduces demographic and socioeconomic disparities^19^. Although studies have been conducted in primary health care settings in Uganda, there is need to further understand malaria health seeking behaviours in communities including among children and pregnant women. This study therefore assessed the malaria health seeking practices including for children under five years of age and pregnant women in Wakiso district, Uganda.

## Methods

### Study design and data collection

The study was cross-sectional and collected quantitative data. The data was collected using a structured questionnaire focusing on: socio-demographic characteristics; malaria health seeking practices for children (when a child was taken to seek healthcare when suspected of being ill, where healthcare was sought for the children, distance travelled to seek healthcare, and whether treatment was paid for); general household health seeking practices (nearest HCF to the home, the distance of the nearest HCF to the home, mode of transport to the nearest HCFs, whether the nearest HCF was used when a household member was suspected of having malaria and reasons for non-use, knowledge of the existence of CHWs in the village and whether they had malaria medicine, and missing of school by children due to malaria); and knowledge and practices on IPTp (knowledge of drug used for IPTp, taking drugs for IPTp for the last pregnancy and reason for not doing so, and number of times they took drugs for IPTp during the last pregnancy). The household questionnaire was administered once for each household involved in the study. The study participants were household heads or other responsible adults such as spouses that were found at home during data collection. However, questions concerning uptake of IPT were asked to only female participants. For this reason, the questionnaire used in the study had a section that was specific for only females.

### Study area and sampling

The study was carried out in Ssisa sub-county, Wakiso district, Uganda located in the central region of the country. Wakiso district has 7 health sub-districts with a total of 229 HCFs where 73% are private and serve over 70% of the population. Ssisa sub-county is made up of 11 rural and peri-urban parishes of Bulwanyi, Bweya, Kitende, Nakawuka, Namulanda, Nankonge, Ngongolo, Nkungulutale, Nsaggu, Ssisa and Wamala which were all involved in the study. The population in the sub-county is engaged in various economic and social activities such as agriculture, animal husbandry, petty trading, stone quarrying, brick making and sand mining. Ssisa sub-county has a population of 94,238 and average household size of 3.820. A total of 727 randomly selected households from 29 villages were involved in the study as described in our earlier publication^21^. The number of villages from each parish involved in the study was determined proportionate to the number of households per parish (data obtained from Uganda Bureau of Statistics) which ranged from 1 (Bulwanyi, Nankonge, Ngongolo, Nsaggu, Ssisa and Wamala) to 7 villages (Kitende and Namulanda). Households involved in the study were randomly selected.

### Data management and analysis

Data were entered in SPSS version 10 (Chicago, Illinois, USA) and analysed in STATA version 13.0 (Stata Corp, Texas, USA). Univariate and bivariate statistical tests were employed. Dependent variables of the study were: the place where treatment was sought by households that had a child below five years who was suspected of having malaria in the two weeks preceding the study (either private or government HCFs); and uptake of two or more IPTp doses during their last pregnancy among women who had a child within the preceding five years. Chi-square and Fisher's exact tests were performed to ascertain the independent variables that were associated with the dependent variables. The independent variables included individual (such as gender, age, education, occupation, religion, and knowledge of the cause of malaria) and household characteristics (such as average household income, distance to the nearest facility, and the number of household members).

### Ethical considerations

Ethical approval to conduct the study was obtained from Makerere University School of Public Health Higher Degrees, Research and Ethics Committee (11353). The research was also approved and registered at the Uganda National Council for Science and Technology (SS 3294). Participation in the study was voluntary, and participants provided written informed consent after explaining to them the purpose of the research including the anticipated risks and potential benefits before taking part.

## Results

### Socio-demographic characteristics of participants

Among the participants, 67.8% were female, 60.3% above 30 years of age, and 44.0% had attained secondary education and above. Regarding average monthly household income, over half of the participants (53.7%) earned less than 40 US dollars, while 65.1% had at least one child under five years of age in their household, with 32.7% having two or more children (Table 1).

**Table 1:** Socio-demographic characteristics of participants

Characteristics	Frequency (N = 727)	Percentage (%)
**Gender**		
Male	234	32.2
Female	493	67.8
**Age (years)**		
18 – 29	289	39.8
≥ 30	438	60.3
**Highest level of education**		
None	78	10.7
Primary	329	45.3
Secondary and above	320	44.0
**Occupation**		
Agriculture	235	32.3
Business	249	34.2
Housewife	82	11.3
Unemployed	108	14.9
Others	53	7.3
**Average household monthly income (US dollars)**		
< 40	390	53.7
≥ 40	337	46.4
**Religion**		
Catholic	294	40.4
Anglican	220	30.3
Pentecostal, Seventh Day Adventists, and other	119	16.4
Muslim	94	12.9
**Status of participant in household**		
Not household head	311	42.8
Household head	416	57.2
**Number of household members**		
1 – 3	228	31.4
4 – 5	231	31.8
> 5	268	36.9
**Household size regarding children under five years**		
Did not have at least one child	254	34.9
Had at least one child	473	65.1
**Number of children under five years in the household**		
None	254	34.9
1	235	32.3
≥ 2	238	32.7

### Malaria health seeking practices for children

The majority of participants (91.9 %) said children suspected of having malaria should be taken for treatment on the same day the illness begins. Among the participants, 29.3% had children under five years of age in their household who were suspected of having malaria in the two weeks preceding the survey. Among these households with suspected sick children, treatment was sought mainly from government HCFs (33.7%), private HCFs (30.7%), and CHWs (22.6%). The places where treatment for the children was first sought was mainly private HCFs (28.1%), followed by government HCFs (25.6%), CHWs (3.0%), and others (13.0%). The place where treatment was first sought for the children (government versus private HCF) was associated with participants' age (p < 0.001), education level (p < 0.001) and household income (p = 0.011) (Table 2). Many of the participants (59.8%) travelled 1 kilometre or less to seek treatment for the child. Others travelled between one to three kilometres (22.1%) or more than 3 kilometres (18.1%). A good number of the participants (69.8%) sought treatment for the child on the same day the condition began. Over half of the participants (53.7%) did not pay for the treatment of the children. Those who paid for the treatment spent between 4 – 20 US dollars ($) (36.2%), above 20$ (6.8%) or less than 4$ (3.4%).

**Table 2:** Place where treatment was sought for children under five years with suspected malaria

Characteristic	Government facility	Private facility	p-value
**Gender**			0.928
Male	25 (53.2)	22 (46.8)	
Female	82 (53.9)	70 (46.0)	
**Age (years)**			< 0.001*
18 – 29	32 (37.2)	54 (62.8)	
≥ 30	75 (66.4)	38 (33.7)	
**Education level**			<0.001**
None	12 (75.0)	4 (25.0)	
Primary	62 (65.3)	33 (34.7)	
Secondary and above	33 (37.5)	55 (62.5)	
**Occupation**			0.135
Agriculture	45 (66.2)	23 (33.8)	
Business	29 (46.8)	33 (53.2)	
Housewife	13 (54.2)	11 (45.8)	
Unemployed	13 (44.8)	16 (56.2)	
Others	7 (43.7)	9 (56.2)	
**Household income**			0.011*
≤ $40	67 (62.0)	41 (38.0)	
> $40	40 (44.0)	51 (56.0)	
**Religion**			0.055
Catholic	37 (54.4)	31 (45.6)	
Anglican	42 (65.6)	22 (34.4)	
Pentecostal, Seventh Day Adventist, and other	17 (43.6)	22 (56.4)	
Muslim	11 (39.3)	17 (60.7)	
**Status of participant in** **household**			0.444
Not household head	50 (51.0)	48 (49.0)	
Household head	57 (56.4)	44 (43.6)	
**Number of household** **members**			0.864
1 – 3	13 (50.0)	13 (50.0)	
4 – 5	38 (52.8)	34 (47.2)	
> 5	56 (55.4)	45 (44.5)	
**Hearing messages about malaria through mass** **media (radio, television or newspapers)**			0.076
Did not hear messages	17 (41.5)	24 (58.5)	
Heard messages	90 (57.0)	68 (43.0)	
**Distance to the** **nearest HCFs (kilometres)**			0.993
≥5	8 (53.3)	7 (46.7)	
< 5	91 (53.2)	80 (46.8)	
**Knowledge on the cause of** **malaria**			0.289
Did not know the cause	9 (42.9)	12 (57.1)	
Knew the cause	98 (55.1)	80 (44.9)	

Statistically significant at *p < 0.05 ** Fisher's Exact test

### Household health seeking practices

The nearest HCFs from participants' households were private clinics (39.3%), government HCFs (30.9%), and private hospitals (20.1%). Other facilities mentioned by participants were pharmacies/drug shops (4.4%). Many of the nearest HCFs (62.3%) were less than one kilometre from the participants' homes. The other facilities were between one to three kilometres (17.2%) or more than three kilometres (10.9%). The most likely ways in which the participants would go to the nearest HCF from their households were walking (65.7%), use commercial motorcycle (28.2%), public transport (3%), private car (1.5%), or bicycle (1.2%). The majority of participants (71.7%) used the nearest HCF from their household the last time a household member was suspected to have malaria. Among participants whose households did not use the nearest facility, the reasons given were suspicion of no drugs being available (33.3%), services being costly (29.8%), poor services offered (18.2%), services needed not offered (8.6%), or poor health worker attitudes (1.5%). A good number of participants (68.4%) were aware of the existence of CHWs who distribute malaria medicines in their villages. However, among participants who were aware of the existence of CHWs, only 17.3% thought they had malaria medicines at the time. In 37.3% of households, school going children had missed school in the previous 6 months due to malaria, with a mean number of days missed of 15.3 (standard deviation ± 29.9).

### Knowledge and practices on Intermittent Preventive Treatment in pregnancy

Over half of the participants (54.2%) were aware of sulfadoxine-pyrimethamine (SP) as the drug used for IPTp. Other drugs participants mentioned were chloroquine (39.3%) and Coartem (6.5%), while 39.3% did not know. Females were significantly more knowledgeable about IPTp compared to males (p < 0.001). Among the females involved in the study (n = 493), 87% had children. The majority of females with children (81.9%) underwent IPTp when they were last pregnant. The main reasons given for those who did not undertake IPTp during their last pregnancy were: not being aware of the need (51.3%); no HCF being available (24.4%), and did not want (16.7%). Among those who took drugs for IPTp, 73.6% took SP, 4.8% took chloroquine, while 16.2% did not know what they took. The majority of women (94.6%) got IPTp the last time they were pregnant during ANC visits while for the rest, it was during another visit to a health facility. Among women who had a child in the five years preceding the study, 179 (63.0%) had obtained two or more IPTp doses during their last pregnancy. Uptake of two or more IPTp doses was associated with higher education level (p = 0.006), having heard messages about malaria through mass media (p = 0.008), as well as knowing the recommended number of IPTp doses (p < 0.001) and drug used in IPTp (p < 0.001) concerning the women (Table 3).

**Table 3:** Association between uptake of IPTp with individual and household characteristics

Variables	Uptake of 2 or more IPTp doses		p-value
	Yes	No	
Overall (n = 284)	179 (63.0)	105 (37.0)	
**Age (years)**			0.577
18 – 29	110 (64.3)	61 (35.7)	
≥ 30	69 (61.1)	44 (38.9)	
**Education level**			0.006*
None	6 (30.0)	14 (70.0)	
Primary	82 (64.1)	46 (35.9)	
Secondary and above	91 (66.9)	45 (33.1)	
**Occupation**			0.417
Agriculture	44 (55.7)	35 (44.3)	
Business	56 (65.9)	29 (34.1)	
Housewife	41 (70.7)	17 (29.3)	
Unemployed	32 (62.7)	19 (37.2)	
Others	6 (54.5)	5 (45.4)	
**Average monthly household income (US dollars)**			0.198
≤ $40	83 (59.3)	57 (40.7)	
> $40	96 (66.7)	48 (33.3)	
**Religion**			0.112
Catholic	79 (68.7)	36 (31.3)	
Anglican	42 (52.5)	38 (47.5)	
Pentecostal, Seventh Day Adventist, and others	36 (67.9)	17 (32.1)	
Muslim	22 (61.1)	14 (38.9)	
**Status of participant in household**			0.931
Not household head	127 (62.9)	75 (37.1)	
Household head	52 (63.4)	30 (36.6)	
**Number of household members**			0.07
1 – 3	46 (71.9)	18 (28.1)	
4 – 5	71 (65.7)	37 (34.3)	
> 5	62 (55.4)	50 (44.6)	
**Household size regarding children under five years**			0.177
Did not have at least one child	7 (46.7)	8 (53.3)	
Had at least one child	172 (63.9)	97 (36.1)	
**Hearing messages about malaria through mass media** **(radio, television or newspaper)**			0.008*
Did not hear messages	36 (50.0)	36 (50.0)	
Heard messages	143 (67.4)	69 (32.5)	
**Distance to the nearest HCF (kilometres)**			0.336
≥ 5	16 (72.7)	6 (27.3)	
< 5	146 (62.4)	88 (37.6)	
**Knowledge on the cause of malaria**			0.497
Did not know the cause	16 (57.1)	12 (42.9)	
Knew the cause	163 (63.7)	93 (36.3)	
**Knowledge on recommended number of IPTp doses**			<0.001*
Did not know the recommended number	59 (45.0)	72 (54.9)	
Knew the recommended number	120 (78.4)	33 (21.6)	
**Knowledge on drug used for IPTp**			<0.001*
Did not know the drug	10 (21.7)	36 (78.3)	
Knew the drug	169 (71.0)	69 (28.9)	

Statistically significant at *p < 0.05

## Discussion

This study explored health seeking practices among under five children on malaria related care as well as uptake of IPTp in Wakiso district, Uganda. Findings indicate that nearly 70% of caretakers of children suspected with malaria sought health care on the day of onset of symptoms, and most of them traveled 3 kilometres or less to the nearest HCF from their home. The choice of facility (government versus private) for seeking malaria related care for children was influenced by the participant's age, education level and household income. Uptake of IPTp among women who had a child within five years preceding the study was generally low (63%) and influenced by level of education, having heard messages about malaria on mass media, as well as knowledge of the drug used during IPTp and recommended number of doses. The findings from our study emphasize the need for improving malaria health seeking practices including IPTp for better health outcomes among the population.

Early diagnosis and appropriate treatment of malaria is critical to minimize severe cases and associated mortality as well as interrupt transmission^22^. In our study, a high number of participants (91.9%) indicated that children with suspected malaria ought to obtain medical care on the day of onset of symptoms. However, only 69.8% of those who had children with suspected malaria reported seeking care on the first day of onset of symptoms. The proportion of children suspected with malaria receiving care on the first day in our study is higher than that reported in a study in Equatorial Guinea (47%)^23^ but lower than that reported in a Kenyan study (86%)^24^. In our study, most caretakers first sought care for the children suspected of having malaria from formal health facilities (private – 28.1%, government - 25.6%) contrary to commonly observed trends in many African settings with high malaria transmission where people tend to manage malaria from home even where biomedical and health services are widely available^23^,^25^. A high number of caretakers seeking malaria health care for children from CHWs is expected in many low-and middle- income country (LMIC) settings where iCCM is being implemented as was established in our study (22.6%). Involvement of CHWs in malaria management among children improves timely access to treatment hence a significant reduction in malaria infections and severe forms^26^. Although these CHWs are usually well known in the community, only 17.3% of participants in our study thought they had malaria medicines at the time. Indeed, lack of medicines among CHWs especially for malaria treatment continues to be an impediment of community programmes aimed at combatting the disease^27^. The Ministry of Health (MOH) should therefore ensure CHWs have the necessary logistics particularly medicines to enable them carry out malaria treatment amongchildren as a form of extending services to the community and reducing the burden of patients on HCFs.

A large proportion of participants would need to travel less than one kilometre (62.3%) or between one to three kilometres (17.2%) to the nearest HCF from their homes. A short distance to a HCF implies that physical access for health care is less of a challenge hence increasing the likelihood of utilizing health services. Evidence suggests that long distances to HCFs impedes utilisation of essential services as has been previously observed in Uganda^27^,^28^. Our study established that more caretakers visited public HCFs (33.7%) than private ones (30.7%) for children suspected of having malaria. However, the first source of treatment for such children was private HCFs (28.1%) compared to public facilities (25.6%). These findings suggest that urgent care for children is sought from private HCFs which are many times more in number and nearer to households than public ones. Nevertheless, public HCFs were still visited especially if the private ones were unable to treat the sick children. However, access to healthcare in public HCFs is impeded by drug stockouts, high staff absenteeism, and long waiting hours^28^,^29^ which sometimes lead to seeking care from private providers. Our study established an association between income and the place where treatment was sought for children suspected of having malaria. This finding is in line with evidence which indicates that utilisation of private health care services increases with rise in education^30^,^31^,^32^. People with higher education are expected to be better informed about health care and usually have higher incomes hence they can afford private health services which sometimes could be of a higher quality than public HCFs^33^. Availability of various options of health services including both private and public facilities in communities is likely to increase access to care for malaria among children.

Regarding uptake of IPTp, 63.0% of women who had given birth in the five years preceding the study had obtained two or more IPTp doses during their last pregnancy. This level of utilisation of IPTp was lower than the national target of 80% for most African countries including Uganda^1^,^34^. The WHO has recommended three or more doses of IPTp for all pregnant women which many countries including Uganda are struggling to achieve1. Despite a low uptake of IPTp in our study, 94.6% got it during ANC the last time they were pregnant. This finding is an indication that low IPTp could be related to low utilisation of ANC as has been found in other studies^35^,^36^. In addition, late timing of the first ANC visit has also been found to contribute to low uptake of IPTp^37^. Regular ANC attendance can improve uptake of optimal doses of IPTp hence sould be encouraged by various stakeholders including the MOH and partners.

Our study established that education was associated with uptake of IPTp among women who had a child five years preceding the study. Having attained higher education is likely to be associated with awareness of malaria prevention programmes. Indeed, individuals with higher education are more likely to be able to understand the benefits of IPTp for maternal and child survival as well as access ANC services^34^. Other studies have also highlighted the effect of education on increased uptake of IPTp^38^,^39^ hence an important factor in malaria prevention in pregnancy. Programmes aimed at increasing awareness on IPTp are therefore likely to particularly benefit women with low levels of education. Knowledge of the recommended number of doses as well as the drug used for IPTp were also associated with uptake of two or more doses. It is possible that this knowledge was because of uptake of IPTp in the first instance given health education is many times carried out during ANC. However, it is not possible to confirm causation given our study was cross-sectional. Nevertheless, other studies have indicated that limited understanding of IPTp among pregnant women affected uptake^40^,^41^. However, knowledge alone may not lead to improved practices regarding IPTp especially with various health system challenges in many LMICs including Uganda such as long distance to HCFs and regular stock out of medicines^28^.

This study had a number of strengths such as involving both women and men in IPTp issues; explored health seeking practices for children, women and the general household; and also involved several parishes that had rural and peri-urban populations. Involving men in IPTp is particularly of key public health importance as male involvement has been shown to contribute to better maternal health outcomes including in developing countries 42. Nevertheless, the study had some limitations such as malaria among children being suspected (as opposed to being actual). In addition, there was potential for recall bias since the study involved asking women questions concerning their pregnancy in the previous five years. However, the specific information that was asked related to only their last pregnancy which for many was within the previous few years hence reducing recall bias. In addition, information concerning the last pregnancy is likely to be known most by women in comparison with earlier pregnancies. Lastly, only univariate and bivariate analysis for associations were done hence confounding could not have been controlled for. These limitations notwithstanding, the findings of this study can inform interventions to improve malaria health seeking practices as well as guide future research in Uganda and beyond.

## Conclusion

This study provides useful insights into malaria health seeking practices including delays in taking children suspected of having the disease to health facilities, and low coverage of IPTp. There is need to improve health malaria seeking practices among childrn and pregnant women to reduce morbidity and mortality related to the disease. Programmes should therefore be designed to increase awareness on the importance of early seeking for health care for children and IPTp, as well as address other health system challenges affecting health seeking behaviour among the community.
